# Predicting accumulation and age at onset of amyloid-β from genetic risk and resilience for Alzheimer's disease

**DOI:** 10.1016/j.ebiom.2026.106329

**Published:** 2026-06-12

**Authors:** Eleanor K. O'Brien, Timothy Cox, Shane Fernandez, Pierrick Bourgeat, Tenielle Porter, Ben Goudey, James D. Doecke, Colin L. Masters, Jurgen Fripp, Kwangsik Nho, Victor L. Villemagne, Carlos Cruchaga, Christopher C. Rowe, Andrew J. Saykin, Vincent Doré, Simon M. Laws

**Affiliations:** a Centre for Precision Health, Edith Cowan University, Joondalup, Western Australia, Australia; b Collaborative Genomics and Translation Group, School of Medical and Health Sciences, Edith Cowan University, Joondalup, Western Australia, Australia; c Australian e-Health Research Centre, CSIRO, Parkville, Victoria, Australia; d Australian e-Health Research Centre, CSIRO, Herston, Queensland, Australia; e Curtin Medical School, Curtin University, Bentley, Western Australia, Australia; f Australia BioCommons, The University of Melbourne, North Melbourne, Victoria, Australia; g Florey Institute, The University of Melbourne, Parkville, Victoria, Australia; h Indiana Alzheimer's Disease Research Center, Indiana University School of Medicine, Indianapolis, IN, USA; i Department of Psychiatry, University of Pittsburgh, Pittsburgh, PA, USA; j Department of Psychiatry, Washington University, St. Louis, MO, 63110, USA; k NeuroGenomics and Informatics, Washington University, St. Louis, MO, 63110, USA; l Department of Molecular Imaging & Therapy and Centre for PET, Austin Health, Heidelberg, Victoria, Australia; m Center for Neuroimaging, Department of Radiology and Imaging Sciences, Indiana University School of Medicine, Indianapolis, IN, USA

**Keywords:** Alzheimer's disease, Amyloid beta accumulation, Age at onset of amyloid beta, Polygenic scores, Risk, Resilience

## Abstract

**Background:**

Accumulation of brain amyloid beta (Aβ), a key pathological hallmark of Alzheimer's disease (AD), begins decades before cognitive symptoms. Being able to predict the risk of Aβ accumulation, or the age at which Aβ exceeds a critical threshold, may enable intervention to delay or prevent onset of AD.

**Methods:**

Using published genome-wide association studies (GWASs), we developed polygenic scores (PGS) for AD risk (PGS_risk_) and resilience (PGS_resilience_), and tested whether these predicted (i) if an individual is an Aβ accumulator (‘Accumulator Status’), and (ii) in accumulators, the age at which brain Aβ exceeds a 20 centiloid (CL) threshold (‘Age at onset of Aβ’; AAO-Aβ) in 2175 participants (1158 with AAO-Aβ) from the Alzheimer's Dementia Onset and Progression in International Cohorts (ADOPIC) study. We also performed GWASs on these traits to develop phenotype-specific PGSs.

**Findings:**

Higher genetic risk of AD predicted increased odds of Aβ accumulation (OR = 1.16; 95% CI = 1.05–1.29; p = 0.003) and younger AAO-Aβ (β = −1.32; SE = 0.31; p = 1.63 × 10^−5^). Higher genetic resilience to AD predicted later AAO-Aβ (β = 0.91; SE = 0.29; p = 0.002) but did not predict Aβ accumulation. These associations were independent of *APOE* ε4 status, the strongest genetic risk factor for AD. Phenotype-specific PGSs were not significantly associated with either trait.

**Interpretation:**

Polygenic scores, alongside other risk factors, may help identify individuals at risk of accumulating Aβ, and predict the age at which this exceeds a critical threshold. This could provide a window for administering disease-modifying treatment or lifestyle interventions to prevent or delay the onset of AD.

**Funding:**

10.13039/100000002National Institutes of Health (R01-AG058676-01A1) and Australian 10.13039/501100000925National Health and Medical Research Council (GNT1161706; GNT2001320).

**Research in context**

**Evidence before this study**

We searched PubMed (last search May 2026) for peer-reviewed studies investigating the genetic basis of Alzheimer's disease (AD), polygenic risk, and genetic predictors of AD-related pathology, using the terms: “Alzheimer∗”, “genetic risk”, “polygenic score∗”, “GWAS”, “amyloid”, “Aβ PET”, “amyloid accumulation”, and “age at onset”. No language or date restrictions were applied.

Genome-wide association studies have identified more than 80 genetic variants associated with AD risk, with the *APOE* ε4 allele being the strongest known genetic determinant. Evidence suggests partial overlap between the genetic architecture of AD risk and that of brain amyloid-β (Aβ) accumulation, a defining pathological hallmark of AD. *APOE* ε4 carriage is associated with greater age-adjusted Aβ burden, and several studies have explored polygenic scores (PGSs) for AD risk or resilience. However, no prior studies have directly evaluated whether polygenic risk or resilience, beyond *APOE* ε4, predicts the age at which Aβ accumulation crosses a pathological threshold, nor whether genetic “resilience” corresponds to a delay in Aβ deposition.

**Added value of this study**

This study uses longitudinal data from the Alzheimer's Disease Onset and Progression in International Cohorts (ADOPIC) study. ADOPIC includes 2175 participants with two or more Aβ positron emission tomography (PET) scans, making it the only harmonised dataset of this scale permitting robust estimation of Aβ accumulation trajectories. This enabled identification of those who were accumulating brain Aβ, and in accumulators, the precise estimation of the age at which Aβ exceeds pathological thresholds.

We were then able to test whether these traits are associated with PGSs capturing genetic risk or resilience for AD. Higher AD polygenic risk was associated with both an increased likelihood of being an accumulator of Aβ and an earlier age at pathological onset of Aβ, whereas higher genetic resilience to AD was associated with a later age at onset. Crucially, these associations persisted after adjusting for *APOE* ε4, indicating independent predictive value of genome-wide polygenic architecture.

**Implications of all the available evidence**

Together with clinical indicators, polygenic scores for AD risk and resilience may improve early identification of individuals likely to exhibit accelerated Aβ accumulation or earlier Aβ pathology onset. Because Aβ deposition begins decades before the emergence of cognitive symptoms, such genomic predictors could help stratify individuals for early interventions, both pharmacological and lifestyle-based, aimed at delaying or preventing the onset of clinical symptoms of AD. These findings support growing evidence that genome-wide polygenic influences shape the earliest detectable stages of AD pathophysiology, offering a potential tool for precision-health approaches to AD prevention.

## Introduction

Alzheimer's disease (AD) is a neurodegenerative disorder, characterised by progressive cognitive decline and memory loss.^1^ It is the most common form of dementia, accounting for around 65% of cases.^2^ The accumulation of amyloid beta (Aβ) in the brain is one of the key pathological hallmarks of AD, beginning years, if not decades, before the onset of clinical symptoms.^3^, ^4^, ^5^ The ‘amyloid cascade hypothesis’ posits that this accumulation precipitates a cascade of events, including tau hyperphosphorylation and formation of neurofibrillary tangles, neuronal loss, cognitive decline and ultimately dementia.^5^^,^^6^ Predicting whether an individual will accumulate Aβ and the age at which they will reach a critical threshold for brain Aβ that leads to these downstream events could therefore enable timely therapeutic strategies aimed at delaying or preventing the onset and progression of cognitive decline.^7^

Late onset AD (LOAD) occurs sporadically in people aged over 65 years, and makes up 90–95% of AD cases.^8^ LOAD (henceforth referred to as AD) is a complex disease, with risk determined by a combination of genetic, environmental and lifestyle factors.^9^ Among the genetic factors, the Apolipoprotein E (*APOE*) gene on chromosome 19 shows the strongest association with the incidence of AD, with the *APOE* ε4 allele conferring a dose-dependent increase in risk of AD, and the ε2 allele being protective.^2^^,^^10^ *APOE* genotype is also associated with the magnitude and timing of Aβ accumulation, such that ε4 carriage is associated with higher average age-adjusted Aβ burden and younger ages when critical Aβ thresholds are reached.^11^ However, genome-wide association studies (GWASs) have identified more than 80 additional genetic variants that show a significant association with AD risk, albeit with smaller individual effects than *APOE*.^12^, ^13^, ^14^, ^15^, ^16^, ^17^, ^18^ GWASs for Aβ accumulation and burden have also identified several significantly associated loci in addition to those within the *APOE* region.^19^, ^20^, ^21^ These findings suggest that the prediction of AD and related traits can be improved by considering genetic factors beyond *APOE*.

Polygenic scores (PGS; also referred to as ‘genetic scores’ or ‘polygenic risk scores’) aggregate the effects of variants across the genome that are associated with the disease or trait of interest.^22^, ^23^, ^24^, ^25^ This approach recognises that complex traits are often influenced by many genetic variants, most of small effect, and many of which will not meet the stringent threshold for genome-wide significance.^26^, ^27^, ^28^ The resulting score gives an estimate of genetic susceptibility to a trait or disease, which may then be used, alongside other clinical indicators, to inform decisions around preventative actions or interventions.^24^ PGSs developed from genetic associations with disease incidence may also be useful for predicting endophenotypes linked to that disease (or *vice versa*), which can provide further insights into the underlying biological disease mechanisms and aid in earlier diagnosis. In the context of AD, genetic risk of disease (as estimated from GWASs for incidence of AD) may therefore be useful for predicting onset and progression of pathophysiological features such as Aβ that occur earlier in the disease trajectory, prior to diagnosis.

In the present study, we develop PGSs based on previously published GWASs for risk of and resilience to AD.^13^^,^^29^ Here, ‘resilience’ was defined as remaining cognitively unimpaired in the face of high genetic risk for AD.^29^ We test the extent to which these PGSs predict whether an individual accumulates brain Aβ and the age at which Aβ is estimated to reach a critical threshold. We then assess whether we can improve on these predictions using *a priori* GWASs to develop phenotype-specific PGSs for each of these traits. We seek to develop predictive models that can identify individuals at high risk for early Aβ pathology, to inform personalised approaches to the prevention and treatment of AD.

## Methods

### Study population

The study included data for 2175 participants from the Alzheimer's Dementia Onset and Progression in International Cohorts (ADOPIC) study, which combines three longitudinal cohort studies: the Alzheimer's Disease Neuroimaging Initiative (ADNI),^30^, ^31^, ^32^ the Australian Imaging, Biomarkers and Lifestyle Study of Ageing (AIBL),^33^^,^^34^ and the Knight ADRC Open Access Series of Imaging Studies (OASIS).^35^ Full details of these studies, including recruitment criteria and the schedule of assessments have been described previously.^30^, ^31^, ^32^, ^33^, ^34^, ^35^ The present study was limited to ADOPIC participants of European ancestry.

### Ethics

The ADNI, AIBL and OASIS studies have all been granted approval by the ethics committees of their respective member institutions. All participants in this study provided informed written consent, and studies were conducted in accordance with the Declaration of Helsinki.

### Genotyping and imputation

DNA was extracted from whole blood samples. For ADNI and AIBL participants, this was done using QIAamp DNA blood spin column kits (Qiagen, Valencia, CA, USA),^31^^,^^33^^,^^34^ and for OASIS participants using the Autogen FlexSTAR + salt precipitation.^36^

ADNI samples were genotyped at genome-wide single nucleotide polymorphisms (SNPs) on either the Illumina Human610-Quad BeadChip or the Illumina Human OmniExpress BeadChip.^31^ AIBL samples were genotyped on the Axiom Precision Medicine Diversity Array (Applied Biosystems™).^33^^,^^34^ OASIS samples were genotyped on one of nine arrays (Illumina Human660W-Quad, Infinium OmniExpressExome-8, Illumina Omni1-Quad, Illumina Human1M-Duo, Infinium Neuro Consortium Array, Infinium CoreExome-24, Infinium Global Screening Array-24, Illumina Human610-Quad, and UK Biobank Axiom array).^36^

**Fig. 1:**
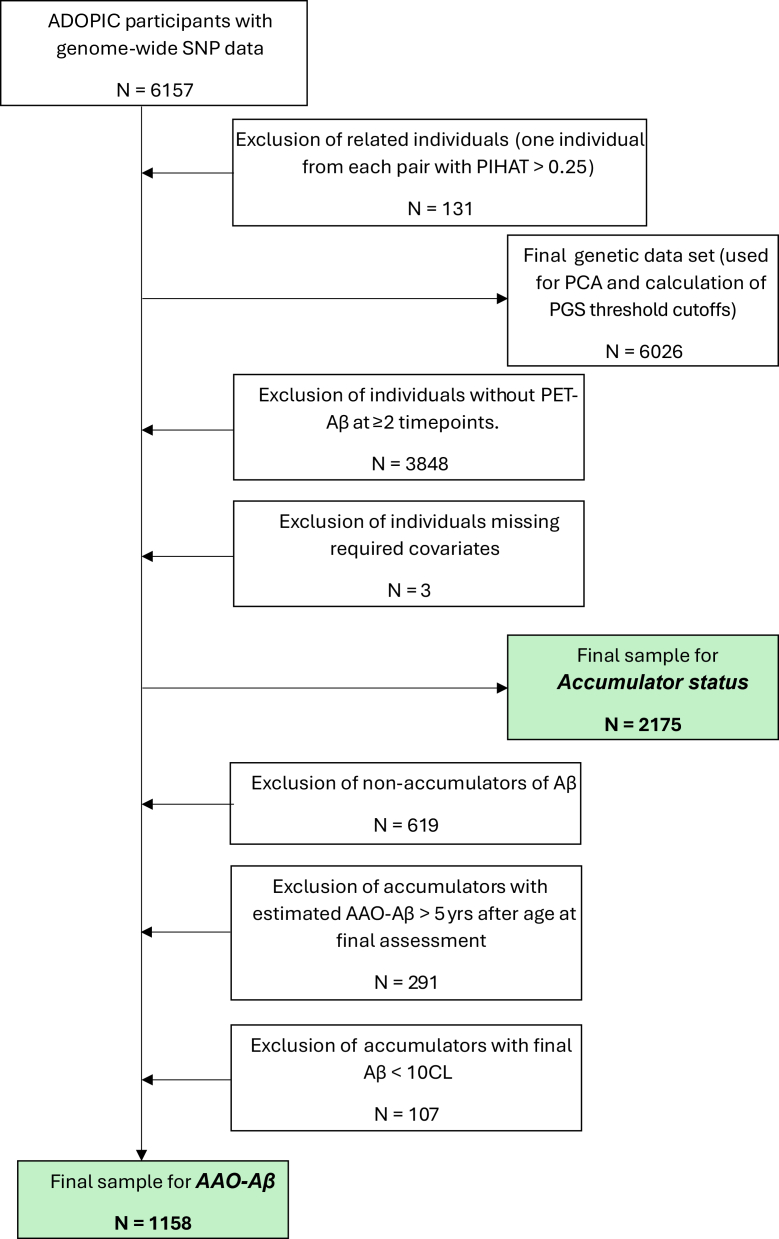
**Sample selection from the ADOPIC study**.

All genotype data were imputed against the TOPMed panel on the TOPMed Imputation Server (University of Michigan, USA).^37^^,^^38^ Within cohorts, this was done for each genotyping run, and then the imputed data were combined across the three cohorts to produce a single harmonised genetic data set. Only autosomal SNPs were included in the final genetic data set. We removed SNPs with an imputation quality r^2^ ≤ 0.3, samples and SNPs with >2% missing genotypes after merging, as well as SNPs with minor allele frequency (MAF) < 5%, and those where the p-value from a test for Hardy–Weinberg equilibrium was <1 × 10^−6^. We used PLINK^39^^,^^40^ to estimate relatedness (pihat) between all pairs of participants and removed one member of each pair of individuals with pihat >0.25. We also used PLINK to conduct a principal components analysis (PCA) on all remaining unrelated individuals to obtain principal components (PCs) to include as covariates to control for population structure in genetic analyses (see below). Genetic data were available for 6157 individuals, of whom 131 were removed due to relatedness, giving a final genetic data set of 6026 individuals (Fig. 1). *APOE* genotype was determined from TaqMan® genotyping assays (Life Technologies, USA) at two SNPs: rs7412 (Assay ID: C__904973_10) and rs429358 (Assay ID: C__3084793_20).

### Aβ PET imaging

Measures of brain β-amyloid (Aβ) burden were obtained for participants in all three cohorts using positron emission tomography (PET) imaging with one of the following five tracers: ^11^C–Pittsburgh compound B (PiB), ^18^F-NAV4694 (NAV), ^18^F-florbetaben (FBB), ^18^F-florbetapir (FBP), or ^18^F-flutemetamol (FLUTE). PET images were analysed using CapAIBL^41^ to generate tracer-specific standardised uptake value ratios (SUVRs), which were then transformed to centiloids (CL) as previously described.^42^^,^^43^ All participants included in this study had at least two Aβ PET scans, with a minimum inter-scan interval of six months. Harmonisation of CL quantification across the three cohorts has been described previously.^44^

### Traits

*Accumulator Status* was determined for all individuals in the study with Aβ PET imaging at two or more timepoints (N = 2175; Fig. 1). Individuals were classified as “accumulators” if they had Aβ PET ≥20 CL at any assessment or showed an increase in Aβ PET across timepoints of ≥0.05 CL/year, and as “non-accumulators” otherwise.^45^

*Estimated Age at Onset of Aβ* (AAO-Aβ) was the age at which participants' cortical Aβ burden was estimated to have reached 20 CL, obtained by placing them on a natural history curve of Aβ accumulation.^45^ A natural history curve for Aβ accumulation was constructed using established methods,^46^ then each participants' baseline time since 20 CL was estimated by finding the value that minimised the squared residuals between the longitudinal data and the natural history curve. The estimated AAO-Aβ was the participants’ baseline age minus their estimated baseline time since 20CL. AAO-Aβ was estimated for people classified as “accumulators” who also met the following criteria: (i) Their Aβ burden at the final scan was ≥10 CL (due to uncertainty around whether people with brain Aβ below this level will reach the 20 CL threshold), and (ii) if brain Aβ did not already exceed the 20 CL threshold, it was predicted to do so within 5 years. In total, AAO-Aβ was estimated for 1158 individuals (Fig. 1).

### Statistics

#### Development of cross-trait polygenic scores

We obtained summary statistics from genome-wide association studies (GWASs) for AD risk^13^ and resilience.^29^ We then used PRSice-2^47^^,^^48^ to identify optimal PGSs for each trait (Accumulator Status and AAO-Aβ), based on risk and resilience GWASs. This process finds the p-value threshold for variant selection that results in a set of variants that explains the largest proportion of variance in the target phenotype, and uses these to create a PGS. To determine whether these risk and resilience PGSs could account for significant variation in each trait beyond that explained by *APOE* (the gene most strongly associated with AD, located on chromosome 19), we excluded chromosome 19 from the genetic data set, and included number of *APOE* ε4 alleles as a covariate, as recommended by Ware et al. (2020).^49^ Additional covariates were sex and study (which of the three cohort studies the participant came from). Age was also included as a covariate in analyses of Accumulator Status. To control for population structure, we included the first three principal components from the PCA as covariates in models for both traits, based on examination of a scree plot of eigenvalues. We used an additive genetic model with default clumping and thinning parameters (250 kb distance, r^2^ threshold 0.1). PGSs were standardised to a mean of 0 and standard deviation of 1.

Four PGSs were derived, defined by the GWAS summary statistics used (risk or resilience) and the trait being predicted (Accumulator Status or AAO-Aβ): PGS_risk-Accumulator_, PGS_resilience-Accumulator_, PGS_risk-AAO_ and PGS_resilience-AAO_. We then used these optimal PGSs (calculated for the whole population) for the relevant trait in (generalised) linear models to test their association with that trait in three groups that were stratified by number of *APOE* ε4 alleles: (a) *APOE* ε4 non-carriers, (b) *APOE* ε4 heterozygotes, and (c) *APOE* ε4 homozygotes. These models included the same covariates as described above, excluding *APOE* ε4 allele count. For Accumulator Status, we fitted logistic regression models using the ‘glm’ function in R with a binomial distribution and logit link function, and for AAO-Aβ we fitted linear regression models using the ‘lm’ function (which assumes a gaussian distribution) in R (version 4.3.3).^50^ In each case, these were implemented in RStudio (version 2024.12.1 Build 563).^51^ Model assumptions were assessed using standard diagnostic procedures, including residual, Q–Q, and leverage diagnostics, Box–Tidwell tests for linearity of continuous predictors on the logit scale, and variance inflation factors (VIFs) to assess multicollinearity. No substantial assumption violations were identified, and all VIFs were <2. Beta coefficients for the PGS term in logistic regressions were exponentiated to convert them to odds ratios (ORs), which represent the change in odds of being an accumulator with each 1 SD increase in the PGS. For linear regressions on AAO-Aβ, beta coefficients for the PGS term represent the change in estimated AAO-Aβ (in years) with a 1 SD increase in the PGS.

To examine the change in odds of being an accumulator and estimated AAO-Aβ between PGS extremes, we also ran models comparing people with scores in the upper and lower quintiles (top and bottom 20%) of the population for each PGS and trait. Threshold PGS scores for the upper and lower quintiles were obtained for all genotyped individuals (N = 6026), and then phenotyped individuals in these ranges were extracted for analysis. In these models, PGS was a categorical predictor with levels “low” (lower quintile) and “high” (upper quintile). Covariates were the same as in models that included the full range of PGS values, and models were again run for all individuals and stratified by number of *APOE* ε4 alleles.

#### GWAS and development of trait-specific PGS using cross-validation

Due to the relatively small size of this study, we used a Monte Carlo cross validation approach^52^^,^^53^ to derive phenotype-specific PGSs. We created 10 cross validation (CV) runs, where within each run the data were split into discovery (two-thirds of individuals; Accumulator Status N = 1450, AAO-Aβ N = 772) and validation (one-third of individuals; Accumulator Status N = 725, AAO-Aβ N = 386) sets using the ‘sample’ function in R (v 4.3.3),^50^ run in R Studio (v 2024.12.1 Build 563).^51^ We conducted a genome-wide association study (GWAS) for the trait of interest in the discovery set and used the summary statistics to find the optimal PGS in the corresponding validation set. This process was repeated in each of the 10 CV runs for each trait.

We ran GWASs for each trait in each discovery data set in PLINK,^39^^,^^40^ using a logistic regression model for Accumulator Status and linear regression for estimated AAO-Aβ. We assumed an additive genetic model, and included sex, study, and the first three principal components as covariates, along with age for Accumulator Status. To identify the optimal PGS for the trait in the corresponding validation set, we used PRSice-2 and followed the same protocol as for the risk and resilience PGSs described above, with the same covariates and clumping parameters, and again with chromosome 19 excluded. Here, the ‘effective allele’ was the allele associated with worse outcomes (higher odds of being an accumulator and earlier estimated AAO-Aβ). Therefore in each case, higher values for these scores are expected to be associated with these worse outcomes. To enable a direct comparison of the performance of the trait-specific PGSs with the risk and resilience PGSs, we tested the association of the optimal risk and resilience PGSs (determined from the whole cohort) with the traits in the validation data set of each CV run, using the same set of covariates as previously.

To compare the predictive performance of each PGS for each trait, we summarised performance across all CV runs. For Accumulator Status, we calculated the mean odds ratio and 95% confidence interval, and for estimated AAO-Aβ, we calculate the mean and standard error of the coefficient of the linear relationship of the standardised PGS against the phenotype across the 10 CV runs. For each PGS and trait, the overall R^2^ value was estimated as the mean of the R^2^ values across CV runs (Nagelkerke's pseudo R^2^ for Accumulator Status), and the overall p-value was estimated from the p-values in each of the 10 CV runs using Stouffer's method,^54^ with p-values weighted by the inverse of the standard error and adjusting for the direction of association in each run.

### Role of funders

The funders had no role in the study design, data collection, analysis, or interpretation, in the writing of the manuscript, or in the decision to submit it for publication.

## Results

### Demographics

**Fig. 2:**
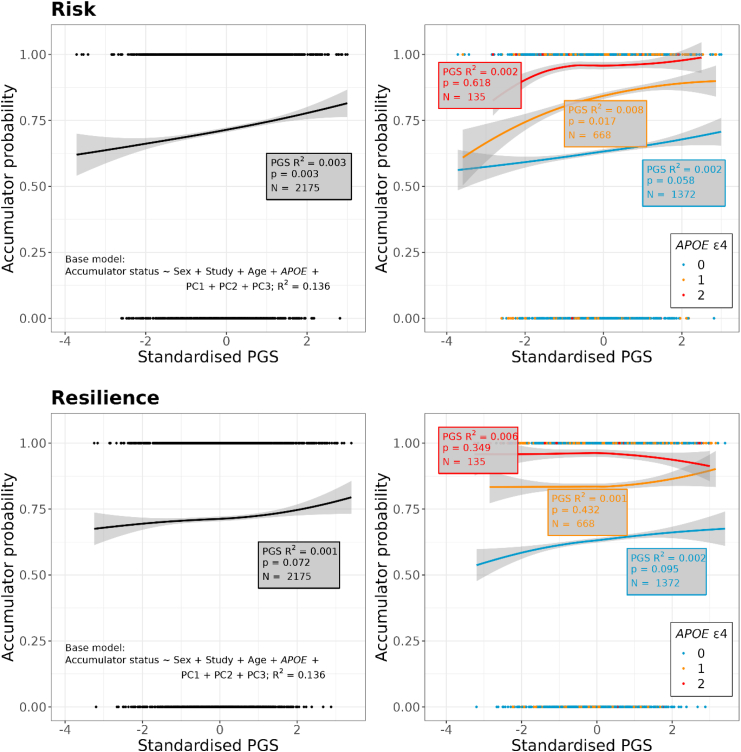
**Plots of standardised polygenic scores (PGS) against probability of being an accumulator of amyloid β**. PGSs were constructed based on genetic risk (top) and resilience (bottom) to Alzheimer's disease (AD). Lines show predicted values from logistic regression models and shading indicates the 95% confidence interval. Plots on the left show the overall relationship for 2175 participants from the ADOPIC study. Plots on the right show the relationship stratified by number of *APOE* ε4 alleles.

**Fig. 3:**
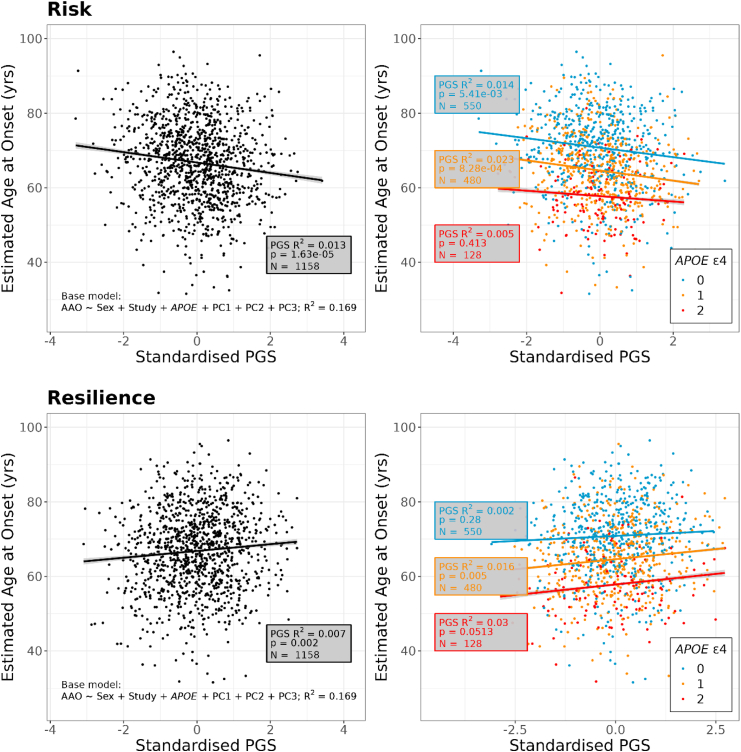
**Plots of standardised polygenic scores (PGS) against estimated age at onset of amyloid β (age when Aβ is estimated to exceed 20 CL)**. PGSs were constructed based on genetic risk (top) and resilience (bottom) to Alzheimer's disease (AD) Lines show predicted values from linear regression models and shading indicates the 95% confidence interval. Plots on the left show the overall relationship for 1158 participants from the ADOPIC study. Plots on the right show the relationship stratified by number of *APOE* ε4 alleles.

Demographic characteristics of all participants with at least two Aβ PET scans (N = 2175), and of the subset of participants with estimated AAO-Aβ (N = 1158), stratified by cohort study, are shown in Table 1. Participants from OASIS were on average younger, a higher proportion of them were female, and there were fewer *APOE* ε4 homozygotes than in ADNI or AIBL. A higher proportion of participants from ADNI were accumulators of Aβ compared to the other cohort studies. In the subset of participants with AAO-Aβ, all of whom were accumulators of Aβ, OASIS participants were still younger and had a lower final Aβ burden (PET Aβ in CL at final assessment used in AAO-Aβ estimation) than those from ADNI and AIBL. OASIS participants had fewer scans and a longer mean inter-scan interval, but a longer mean duration of follow-up (Table 1). Participant counts and percentages by Accumulator Status, for each *APOE* ε4 group, are shown in Table 2. The percentage of participants who were accumulators of Aβ increased with number of *APOE* ε4 alleles, from 63.1% among non-carriers, to 84% of ε4 heterozygotes and 95.6% of ε4 homozygotes (Table 2). Consistent with previous studies, *APOE* ε4 carriage was strongly associated with both Accumulator Status and estimated AAO-Aβ in a dose-dependent manner, with an increasing number of ε4 alleles associated with higher odds of being an accumulator of Aβ (Fig. 2, Table S1) and earlier estimated AAO-Aβ (Fig. 3, Table S2). We also compared the duration of follow up, mean inter-scan interval and number of scans between accumulators and non-accumulators to assess whether they may affect classification. None of these measures differed between the groups (Table S3).

**Table 1:** Summary of demographic characteristics of (a) all participants included in the study and (b) participants that met the criteria for inclusion in analysis of estimated AAO-Aβ, shown overall and stratified by study cohort.

Characteristic	Overall N = 2175	ADNI N = 952	AIBL N = 930	OASIS N = 293	p-value
**(a) All participants**					
**Sex N (%)**					0.003
Female	1100 (50.6%)	450 (47.3%)	479 (51.5%)	171 (58.4%)	
Male	1075 (49.4%)	502 (52.7%)	451 (48.5%)	122 (41.6%)	
Mean follow-up duration yrs (SD)	4.2 (2.6)	4.2 (2.6)	3.9 (2.6)	5.0 (2.4)	<0.001
Mean inter-scan interval months (SD)	26.6 (11.1)	26.2 (8.0)	23.0 (8.8)	39.8 (15.8)	<0.001
Mean no. of PET scans (SD)	3.0 (1.3)	3.0 (1.2)	3.1 (1.5)	2.6 (0.7)	<0.001
**Accumulator status N (%)**					<0.001
Non-accumulator	619 (28.5%)	166 (17.4%)	362 (38.9%)	91 (31.1%)	
Accumulator	1556 (71.5%)	786 (82.6%)	568 (61.1%)	202 (68.9%)	
**Mean final age yrs (SD)**	76.0 (7.7)	77.0 (7.7)	76.3 (6.7)	71.5 (8.9)	<0.001
**No. of *APOE* ε4 alleles N (%)**					0.021
0	1372 (63.1%)	567 (59.6%)	611 (65.7%)	194 (66.2%)	
1	668 (30.7%)	319 (33.5%)	261 (28.1%)	88 (30.0%)	
2	135 (6.2%)	66 (6.9%)	58 (6.2%)	11 (3.8%)	

Characteristic	Overall N = 1158	ADNI N = 649	AIBL N = 400	OASIS N = 109	p-value
**(b) Participants with estimated age at onset of Aβ**					
**Sex N (%)**					0.071
Female	564 (48.7%)	301 (46.4%)	200 (50.0%)	63 (57.8%)	
Male	594 (51.3%)	348 (53.6%)	200 (50.0%)	46 (42.2%)	
Mean follow-up duration yrs (SD)	4.1 (2.6)	4.2 (2.6)	3.8 (2.6)	5.0 (2.5)	<0.001
Mean inter-scan interval months (SD)	26.2 (11.4)	26.2 (8.7)	22.5 (10.0)	40.1 (17.7)	<0.001
Mean no. of PET scans (SD)	3.0 (1.3)	3.0 (1.2)	3.1 (1.5)	2.6 (0.7)	0.011
**Mean final age yrs (SD)**	77.7 (7.3)	77.9 (7.5)	77.9 (6.8)	75.6 (7.8)	0.018
**Mean final PET Aβ CL (SD)**	69.4 (40.0)	71.5 (42.3)	71.3 (37.2)	49.7 (29.0)	<0.001
**Mean estimated AAO-Aβ yrs (SD)**	66.8 (10.8)	66.5 (11.4)	66.7 (10.2)	69.1 (8.8)	0.100
**No. of *APOE* ε4 alleles N (%)**					0.400
0	550 (47.5%)	316 (48.7%)	185 (46.3%)	49 (44.9%)	
1	480 (41.5%)	268 (41.3%)	162 (40.5%)	50 (45.9%)	
2	128 (11.1%)	65 (10.0%)	53 (13.2%)	10 (9.2%)	

P-values are from comparisons between study cohorts using either a Pearson's chi square test (sex, accumulator status, no. of APOE ε4 alleles) or a Kruskal–Wallis rank sum test (final age, final PET Aβ, estimated AAO).

**Table 2:** Counts (%) of individuals classified as non-accumulators and accumulators of Aβ, stratified by number of *APOE* ε4 alleles.

Aβ accumulator status	Number of *APOE* ε4 alleles			Total
	0	1	2	
Non-accumulator	506 (36.9%)	107 (16.0%)	6 (4.4%)	619 (28.5%)
Accumulator	866 (63.1%)	561 (84.0%)	129 (95.6%)	1556 (71.5%)
TOTAL	1372	668	135	2175

Polygenic scores derived from risk and resilience GWAS against Accumulator Status and Estimated Age at Onset of Aβ (AAO-Aβ).

The optimal risk PGS for Accumulator Status (PGS_risk-Accumulator_) was significantly associated with this trait overall, with an odds ratio (OR) of 1.16 (95% CI 1.05–1.29) (Table S1), indicating that for an increase of 1 standard deviation (SD) in PGS_risk-Accumulator_, the odds of being an accumulator of Aβ increase by 16%. When stratified by number of *APOE* ε4 alleles, the same direction of association was seen in all groups, but this was only significant in *APOE* ε4 heterozygotes (marginally non-significant in ε4 non-carriers, p = 0.058) (Fig. 2, Table S1). Individuals in the upper quintile of PGS_risk-Accumulator_ were 63% more likely than those in the bottom quintile to be accumulators of Aβ overall (OR = 1.63; 95% CI 1.18–2.26) (Table S1), and this difference was also significant within *APOE* ε4 non-carriers and heterozygotes (Table S1). The optimal resilience PGS (PGS_resilience-Accumulator_) was not significantly associated with Accumulator Status overall, or for any group when stratified by number of *APOE* ε4 alleles (Fig. 2, Table S1). We also did not detect a significant difference in the probability of being an accumulator between the upper and lower quintiles of PGS_resilience-Accumulator_ (Table S1). It is important to note that only 4.4% (6 out of 135) of *APOE* ε4 homozygotes in our study were non-accumulators (Table 2). This was a much lower percentage than in the other ε4 groups (Table 2), and would have limited our power to detect an association with Accumulator Status in this sub-group. Because the original AD resilience GWAS was conducted in a population of people at high genetic risk,^29^ we conducted follow-up analyses of the relationship of PGS_resilience-Accumulator_ that additionally included PGS_risk-Accumulator_ as a covariate. However, this made no qualitative difference to the results (Table S1).

The optimal risk and resilience PGSs for estimated AAO-Aβ (PGS_risk-AAO_ and PGS_resilience-AAO_) were both significant predictors of this trait overall, with higher PGS_risk-AAO_ and lower PGS_resilience-AAO_ associated with an earlier estimated AAO-Aβ (Fig. 3, Table S2). A 1 SD increase in PGS_risk-AAO_ and a 1 SD decrease in PGS_resilience-AAO_ were associated with estimated AAO-Aβ 1.3 years and 0.91 years earlier respectively (Fig. 3, Table S2). When stratified by number of *APOE* ε4 alleles, higher PGS_risk-AAO_ was associated with an earlier estimated AAO-Aβ in *APOE* ε4 non-carriers and heterozygotes, but there was no significant association with AAO-Aβ in *APOE* ε4 homozygotes. Individuals in the upper quintile for PGS_risk-AAO_ had a mean estimated AAO-Aβ 2.9 years earlier than those in the lower quintile after accounting for other covariates, while among *APOE* ε4 heterozygotes this difference was 4.5 years (Table S2). Higher PGS_resilience-AAO_ was associated with a later estimated AAO-Aβ in *APOE* ε4 heterozygotes and a marginally non-significant (p = 0.051) increase in estimated AAO-Aβ in ε4 homozygotes, but not associated with AAO-Aβ in *APOE* ε4 non-carriers (Fig. 3, Table S2). Individuals in the upper quintile for PGS_resilience-AAO_ had a mean estimated AAO-Aβ 2.2 years later than those in the lower quintile after accounting for other covariates, but there was not a significant difference between these extremes for any of the groups after stratification by number of *APOE* ε4 alleles (Table S2).

There was no overlap in the SNPs included in PGS_risk_ and those in PGS_resilience_ for either trait. This was because in the original construction of the AD resilience GWAS used here,^29^ any SNPs with p < 0.5 in the Kunkle et al. (2019)^13^ AD risk GWAS were excluded to ensure that it captured genetic variation in resilience to AD that was independent of that associated with AD risk.^29^ Within each base GWAS, all SNPs below the optimal p-value threshold identified by PRSice2 after clumping were included in the PGS, meaning that the PGS for the trait with the higher p-value threshold contained all of the SNPs in that with the lower threshold. Therefore, for PGS_risk_, the 10 SNPs in PGS_risk-Accumulator_ (p-value threshold = 5 × 10^−8^; Table S1) were among the 3325 in PGS_risk-AAO_ (p-value threshold = 5.6 × 10^−3^; Table S2), while for PGS_resilience_, the 34 SNPs in PGS_resilience-AAO_ (p-value threshold = 1.75 × 10^−3^; Table S2) were among the 274 in PGS_resilience-Accumulator_ (p-value threshold = 0.014; Table S1).

Polygenic scores derived from trait-specific GWASs against Accumulator Status and Estimated Age at Onset of Aβ (AAO-Aβ), and comparison with risk and resilience PGSs.

The mean percentage of people who were accumulators of Aβ across each of the CV runs was 71.4% in the discovery sets (SD 0.50) and 71.8% in the validation sets (SD 1.00) (Table S4), which are both very close to the value of 71.5% for the whole sample (Table 1). In the trait-specific GWASs run in each of the 10 CV runs (excluding chromosome 19), we found a total of two SNPs (rs12192157 and rs6900289) associated with Accumulator Status at genome-wide significance (p < 5 × 10^−8^). These closely linked variants were on chromosome 6 and were significant in just one of the CV runs (Table S5). We found one genome-wide significant SNP (rs12022131) for estimated AAO-Aβ in one of the CV runs, located on chromosome 1 (Table S5).

The optimal trait-specific PGS for Accumulator Status (PGS_Accumulator_) was significantly associated with this trait in four of the 10 CV runs (two of these remained significant after FDR correction), although the direction of the association varied among runs and the overall association was not significant (Fig. S1, Table S6). The risk and resilience PGSs for Accumulator Status, run in the validation sets of each CV run for comparison, both showed overall significant positive associations with odds of being an accumulator. In contrast to PGS_Accumulator_, the direction of association was consistent across all 10 CV runs for PGS_risk-Accumulator_ and all but one CV run for PGS_resilience-Accumulator_, although it was only significant in six CV runs for PGS_risk-Accumulator_ and not in any individual CV run for PGS_resilience-Accumulator_ (Fig. S1; Table S6).

Similarly, for estimated AAO-Aβ, the optimal trait-specific PGS (PGS_AAO_) was not significantly associated with the trait overall, and although the association was significant in four of the CV runs (three after FDR correction), the direction of association was highly variable across runs (Fig. S1, Table S7). PGS_risk-AAO_ was negatively associated with AAO-Aβ, indicating that individuals with a higher genetic risk of AD had an earlier estimated age at onset of Aβ. PGS_resilience-AAO_ was positively associated with AAO-Aβ, indicating a later estimated age at onset of Aβ in more genetically resilient individuals (Fig. S1, Table S7). Of the three PGSs for each trait, PGS_risk_ had, on average, the largest effect size and explained the greatest proportion of the variation in both Accumulator Status and estimated AAO-Aβ (Fig. S1).

## Discussion

We tested whether polygenic scores based on genetic risk of and resilience to AD could predict Aβ Accumulator Status and estimated AAO-Aβ. We also tested whether PGSs based on phenotype-specific GWASs improved prediction of these traits. Higher genetic risk of AD was associated with higher odds of Aβ accumulation, and earlier estimated AAO-Aβ. Higher genetic resilience was associated with a later estimated AAO-Aβ but was not a significant predictor of Accumulator Status. Phenotype-specific PGSs did not improve on the predictive performance of PGS_risk_ or PGS_resilience_ and were not significant predictors of either trait overall.

The associations of PGSs with each trait were seen after accounting for *APOE* ε4 status, the strongest genetic risk factor for AD.^2^^,^^10^ Our results contrast with several studies that have found that PGSs did not improve prediction over and above *APOE* ε4 status for AD and all-cause dementia (ACD),^55^ or for measures of Aβ deposition.^56^^,^^57^ However, other studies have found that PGSs do result in small but significant improvements over *APOE* alone in prediction of AD-related traits, including incidence of AD^57^^,^^58^ and Aβ pathology.^59^ Apparent inconsistencies between these findings may be explained by variation in the methods used to construct PGSs, as well as the specific phenotypes examined. It has been argued that Aβ deposition is largely driven by *APOE*, and that other genetic contributors to AD become more important at later disease stages.^55^^,^^57^ However, our results highlight that considering genetic factors beyond *APOE* can improve prediction of whether and how early individuals will accumulate Aβ.

Differences between PGS_risk_ and PGS_resilience_ in their associations with the traits examined, and their interactions with *APOE*, offer insights into the mechanisms by which the genetic variation captured by these scores confers risk or protection against AD. Increased genetic risk of AD was a stronger predictor of adverse outcomes (higher odds of being an accumulator and earlier estimated AAO-Aβ) in ε4 non-carriers and heterozygotes than in ε4 homozygotes, suggesting it contributes little additional risk in individuals who are already at highest risk due to *APOE* ε4 homozygosity. By contrast, higher genetic resilience to AD was associated with later estimated AAO-Aβ in ε4 heterozygotes (and a marginally non-significant association in homozygotes), but was not associated with AAO-Aβ in ε4 non-carriers. The original AD resilience GWAS was conducted by limiting the study population to individuals at high genetic risk for AD (defined by a similar risk PGS to that developed here) and contrasting ‘resilient’ (unaffected by AD) individuals with AD cases.^29^ This means that it is effectively a measure of resilience to genetic risk of AD, so it is unsurprising that it is a stronger predictor of AAO-Aβ in *APOE* ε4 carriers, who are at highest genetic risk.^2^^,^^10^ PGS_resilience_ was not associated with Accumulator Status, although the overall trend (significant when aggregated across runs in the cross-validation study) was positive, implying that more genetically resilient individuals are *more* likely to be accumulators of Aβ. While this seems counterintuitive, it is likely another consequence of the fact that for this score, more genetically resilient individuals also have higher genetic risk scores,^29^ although accounting for PGS_risk_ in the analysis did not qualitatively change this result (Table S1). A framework previously proposed when considering protective factors for AD, distinguishes between ‘resistance’ and ‘resilience’, where resistance refers to the avoidance of pathological brain changes, while resilience is the ability to cope with accumulating neuropathology and avoid brain atrophy or cognitive decline.^60^, ^61^, ^62^ The lack of association of PGS_resilience_ with Accumulator Status in our study suggests that genetic variation captured by this score does not confer protection against AD by preventing the accumulation of Aβ (‘resistance’), although the association with AAO-Aβ suggests it may slow or delay this accumulation. Further analysis involving a broader range of traits is required to disentangle this.

The trait-specific PGSs for Accumulator Status and estimated AAO-Aβ were developed to evaluate whether these could improve prediction of these traits over those derived from external GWASs based on AD risk and resilience. However, consistent with expectations given the modest sample sizes available for discovery GWASs (N = 1450 for Accumulator Status and N = 772 for AAO-Aβ, compared with N = 94,437 for risk^13^ and N = 13,572 for resilience^29^), results were highly variable across cross-validation folds and did not yield stable associations. This supports our use of polygenic scores derived from large external GWASs of related traits. Despite the lack of compelling evidence in the current study, trait-specific PGSs may nevertheless capture unique genetic variation associated with these traits, particularly in larger studies. We identified two closely-linked SNPs on chromosome 6 that were associated with Accumulator Status (rs12192157 and rs6900289), and one SNP on chromosome 1 (rs12022131) that was associated with AAO-Aβ at a genome-wide significant level. While each was significant in only a single CV run, it will be of interest to determine whether a signal is seen in these regions in future studies. To the best of our knowledge, there are currently no known associations of these SNPs with specific traits or diseases. However, the SNPs on chromosome 6 are in close proximity to the *LPA* gene, which affects plasma concentrations of lipoprotein (a) (Lp(a)) and is strongly associated with cardiovascular disease,^63^ a key risk factor for AD.^64^^,^^65^

Genetic variants, unlike other biomarkers of disease, remain constant across the lifespan, meaning that polygenic scores for diseases and related traits offer the potential to identify high risk individuals at a very early stage, prior to symptom onset.^25^ In AD, a diagnosis is typically made once cognition is impaired, by which time there has been widespread damage to the brain. However, brain Aβ begins accumulating years or decades prior to appearance of cognitive symptoms.^3^, ^4^, ^5^ Therefore, identifying individuals at risk of accumulating Aβ provides an opportunity to administer interventions to prevent or delay onset and progression of disease. While effective treatments for AD have proved elusive, recent years have seen the development of anti-amyloid monoclonal antibodies that remove Aβ from the brain and have been shown to produce modest slowing of cognitive decline in people with mild symptomatic AD.^66^, ^67^, ^68^ Trials are currently evaluating whether administering these treatments at an earlier stage, in asymptomatic individuals, may be more effective and there is hope that it may eventually be possible to prevent AD.^69^ In this instance, PGSs could offer a relatively inexpensive and minimally invasive method to evaluate people's risk and prioritise them for further screening. A strength of this study is that it develops independent PGSs for Aβ accumulator status and AAO-Aβ. The combination of these provides a framework for predicting both the likelihood of being an Aβ accumulator and the expected age at which Aβ exceeds a critical threshold, conditional on this likelihood. A limitation of PGSs is that they typically explain only a small proportion of the total variation in a disease or trait,^70^ which was the case in this study (0.2–2.3% for PGS_risk_; 0.1–1.6% for PGS_resilience_, depending on population sub-group). While this limits their utility for making a definitive diagnosis, our results show that PGSs can nevertheless improve the accuracy of prediction of AD-related traits, and may be a useful tool for risk stratification, particularly when considered alongside other risk predictors including *APOE* genotype, demographics and lifestyle.

This study does have several limitations. Within our study population, over 70% of participants were accumulators of Aβ. While data on Accumulator Status in the wider population are scarce, one study found that ∼20% of healthy adults aged ≥60 years had elevated Aβ.^71^ Accumulators of Aβ are therefore almost certainly over-represented in our study population, which is unsurprising given that the component cohorts are enriched for people with cognitive complaints.^30^, ^31^, ^32^, ^33^, ^34^, ^35^ As a result, the likelihood of being an accumulator as predicted by PGS score may be overestimated and should be recalibrated based on a more representative population. Similarly, AAO-Aβ could only be estimated for people who had begun accumulating Aβ and were close to, or had exceeded, the 20 CL threshold. The predictive performance of this score in the broader population, including people with low Aβ who may accumulate Aβ, needs to be verified. Furthermore, the lack of an external validation sample for the phenotype-specific PGSs meant that it was necessary to both run the GWASs and develop PGSs within the study population by dividing it into discovery and validation data sets. Despite utilising the largest existing dataset for these traits, this resulted in small sample sizes relative to those used in the GWASs from which risk and resilience PGSs were derived.^13^^,^^29^ This was partially addressed by our cross-validation approach. However, the instability of score performance across CV runs is likely due to the small sample. Finally, the study population was limited to people of European ancestry. Differences in linkage disequilibrium (LD) structure, allele frequencies and genetic architecture can affect the generalisability of genetic predictors across different ancestries,^72^ therefore testing in diverse populations would be beneficial.

Polygenic scores based on genetic risk of AD explained a small but significant proportion of the variation in Accumulator Status and estimated AAO-Aβ, over and above that explained by *APOE* ε4. The PGS for AD risk may be particularly useful, in combination with other predictors, for identifying individuals at risk of Aβ accumulation and earlier AAO-Aβ, who may benefit from targeted prevention and treatment.

## Contributors

EKO, SML and TP conceptualised and designed the study. TC generated the estimates of age at onset of Aβ. All data were accessed and verified by EO, SF and TP. Data on Accumulator Status and AAO-Aβ were additionally verified by TC. EKO developed the methodology for, and performed, the other statistical analyses, with advice and review by BG, SF, TP, JF and JDD. PB, KN, VLV, VD, CC, AJS, TP and SML contributed to acquisition or curation of data. CLM, CCR, CC, AJS and SML contributed to funding acquisition. SML was responsible for the overall supervision of the study. EKO, TP, and SML contributed to the original draft. All authors contributed to the revision and editing of the manuscript and approved the submitted version.

## Data sharing statement

The datasets generated and analysed during the current study are not publicly available due to individual cohort restrictions as outlined at: ADNI: https://adni.loni.usc.edu/data-samples/adni-data/; AIBL: https://aibl.org.au/collaboration/#data-access; OASIS: https://sites.wustl.edu/oasisbrains/home/access/. However, data are available from the corresponding author on reasonable request, with the permission of the participating cohort studies.

## Declaration of interests

CLM has served on the boards of Alzheimer's Research Australia and the CRC for Mental Health, and has undertaken consultancies with Actinogen, Acumen, Alterity, Biogen, Eisai, Eli Lilly, Roche and Sunbird. KN has undertaken consulting for the National Research Foundation of Korea. CC is a member of the scientific advisory board of Circular Genomics and owns stocks, and is on the scientific advisory board of ADmit and Alamar, consults for Sanofi, NovoNordisk, and Owkin, and has received research support from GSK, Danaher and EISAI. All other authors report no competing interests relevant to this manuscript.
