# Supplementary material for: Predicting accumulation and age at onset of amyloid-β from genetic risk and resilience for Alzheimer's disease
Source: eBioMedicine. 2026 Jun 12;129:106329. doi: 10.1016/j.ebiom.2026.106329 (PMC13276526; doi:10.1016/j.ebiom.2026.106329)
Supplement: Figure S1 [file mmc2.pdf]

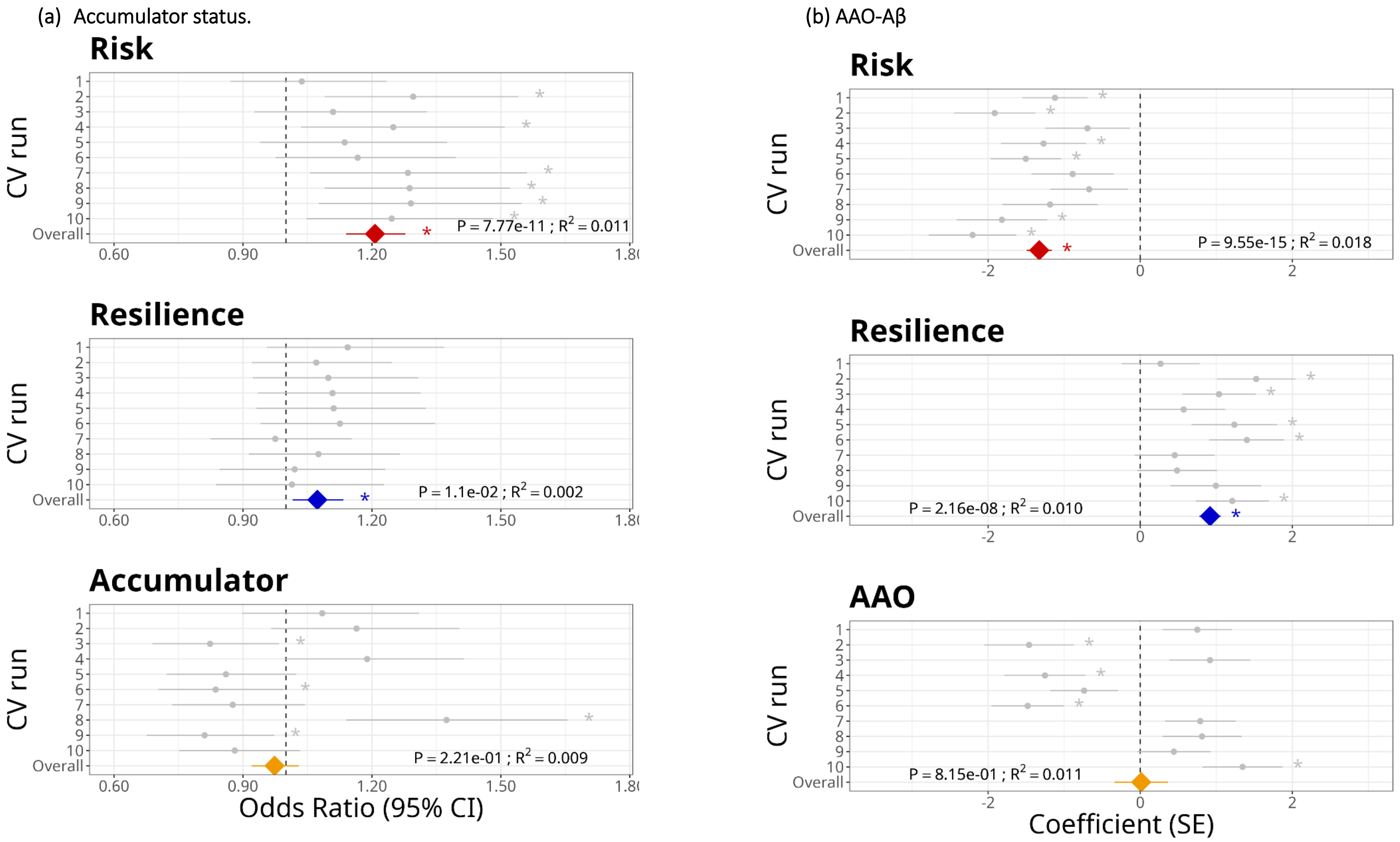

Fig. S1. Forest plots of the relationship of standardised polygenic scores (PGS) with each Aβ trait. (a) Accumulator status (probability of being an accumulator of Aβ) and (b) Estimated AAO-Aβ (age when Aβ is estimated to exceed 20 CL). Three PGSs were evaluated for each trait: risk and resilience to AD based on previously published GWAS, and a phenotype-specific PGS based on an *a priori* GWAS within the current data set, using a cross-validation (CV) approach. Grey dots and lines indicate the odds ratio and its 95 % confidence interval (accumulator status) or the coefficient and standard error of the linear relationship (AAO- Aβ) of the standardised PGS against the phenotype within the validation set of each CV run. Coloured diamonds show the mean odds ratio or coefficient for each PGS across the 10 CV runs. Asterisks indicate a significant ($P < 0.05$) association of PGS with the trait.
